# Patterns of *Midichloria* infection in avian-borne African ticks and their trans-Saharan migratory hosts

**DOI:** 10.1186/s13071-018-2669-z

**Published:** 2018-02-22

**Authors:** Irene Di Lecce, Chiara Bazzocchi, Jacopo G. Cecere, Sara Epis, Davide Sassera, Barbara M. Villani, Gaia Bazzi, Agata Negri, Nicola Saino, Fernando Spina, Claudio Bandi, Diego Rubolini

**Affiliations:** 1 Wild Urban Evolution and Ecology Lab, Centre of New Technologies, University of Warsaw, Banacha 2C, 02-097 Warsaw, Poland; 2 Dipartimento di Medicina Veterinaria, Università degli Studi di Milano, via Celoria 10, I-20133 Milan, Italy; 3 Istituto Superiore per la Protezione e la Ricerca Ambientale (ISPRA), via Cà Fornacetta 9, I-40064 Ozzano Emilia (BO), Italy; 4 Dipartimento di Bioscienze, Università degli Studi di Milano, via Celoria 26, I-20133 Milan, Italy; 5 Dipartimento di Biologia e Biotecnologie, Università degli Studi di Pavia, via Ferrata 9, I-27100 Pavia, Italy; 6 Dipartimento di Scienze e Politiche Ambientali, Università degli Studi di Milano, via Celoria 26, I-20133 Milan, Italy

**Keywords:** Bacteriaemia, Ectoparasites, Endosymbionts, Hard ticks, *Hyalomma*, Horizontal transmission, *Midichloria mitochondrii*, Migratory birds

## Abstract

**Background:**

Ticks are obligate haematophagous ectoparasites of vertebrates and frequently parasitize avian species that can carry them across continents during their long-distance migrations. Ticks may have detrimental effects on the health state of their avian hosts, which can be either directly caused by blood-draining or mediated by microbial pathogens transmitted during the blood meal. Indeed, ticks host complex microbial communities, including bacterial pathogens and symbionts. *Midichloria* bacteria (*Rickettsiales*) are widespread tick endosymbionts that can be transmitted to vertebrate hosts during the tick bite, inducing an antibody response. Their actual role as infectious/pathogenic agents is, however, unclear.

**Methods:**

We screened for *Midichloria* DNA African ticks and blood samples collected from trans-Saharan migratory songbirds at their arrival in Europe during spring migration.

**Results:**

Tick infestation rate was 5.7%, with most ticks belonging to the *Hyalomma marginatum* species complex. Over 90% of *Hyalomma* ticks harboured DNA of *Midichloria* bacteria belonging to the monophylum associated with ticks. *Midichloria* DNA was detected in 43% of blood samples of avian hosts. Tick-infested adult birds were significantly more likely to test positive to the presence of *Midichloria* DNA than non-infested adults and second-year individuals, suggesting a long-term persistence of these bacteria within avian hosts. Tick parasitism was associated with a significantly delayed timing of spring migration of avian hosts but had no significant effects on body condition, whereas blood *Midichloria* DNA presence negatively affected fat deposits of tick-infested avian hosts.

**Conclusions:**

Our results show that ticks effectively transfer *Midichloria* bacteria to avian hosts, supporting the hypothesis that they are infectious to vertebrates. Bird infection likely enhances the horizontal spread of these bacteria across haematophagous ectoparasite populations. Moreover, we showed that *Midichloria* and tick parasitism have detrimental non-independent effects on avian host health during migration, highlighting the complexity of interactions involving ticks, their vertebrate hosts, and tick-borne bacteria.

**Electronic supplementary material:**

The online version of this article (10.1186/s13071-018-2669-z) contains supplementary material, which is available to authorized users.

## Background

Ticks are widespread obligate haematophagous ectoparasites of vertebrates [1]. Upon attachment to vertebrate hosts, ticks may negatively affect their fitness either directly, by draining resources (i.e. blood), thus affecting hosts’ metabolism, by favouring secondary bacterial infections, or by transmitting microbes via the blood meal [1]. Ticks indeed harbour complex microbial communities, comprising agents causing major bacterial, viral and protozoan diseases, with potentially important consequences for both animal and human health [2, 3]. Birds are frequent intermediate or definitive hosts of many tick species [4], and they appear to play a key role for the spread of ticks and tick-borne agents of veterinary and medical relevance over broad geographical areas [5, 6]. During their migratory movements, birds may in fact disperse ticks attached to their bodies (and tick-associated microbial communities) across continents and along migration routes [5, 7]. For instance, Afro-Palaearctic migratory birds are frequently infested with immature stages of African *Hyalomma* ticks while they travel back from African non-breeding areas to their European breeding quarters in spring [8]. In spite of the relatively frequent occurrence of ticks on migrating birds, the effects of tick parasitism on the health state of avian hosts have been mainly evaluated by studies carried out during the reproductive period (e.g. [9–11]), whereas the health effects of tick attachment during migration have received less attention (e.g. [12, 13]). The intensity of negative effects of tick infestation on the health state of avian hosts appears highly variable. Some studies reported increased stress levels, anaemia due to blood loss, hypoproteinaemia, mass loss and low body condition in tick-infested birds compared to non-infested ones [10, 11, 13–16], sometimes leading to increased mortality of heavily infested individuals [10], while others failed to detect significant negative effects of natural levels of tick infestation on health parameters [12, 17, 18].

Besides vertebrate pathogens, tick microbial communities may include arthropod endosymbionts [19]. A case in point is “*Candidatus* Midichloria mitochondrii” (hereafter *M. mitochondrii*), a bacterium that resides in the ovarian cells of adult female *Ixodes ricinus* ticks, as well as in the primordial ovarian tissue of female larvae and nymphs [20]. The bacterium has been detected in the cytoplasm of the oocytes, but also inside mitochondria [21]. *M. mitochondrii* infects 100% of female *I. ricinus*, while male infection rates are lower [21, 22]. In addition, this bacterium has been detected in 100% of eggs, indicating vertical transmission to the progeny [21, 22]. In recent years, many bacteria phylogenetically related to *M. mitochondrii* have been detected in tick species from different genera worldwide (*Ixodes*, *Rhipicephalus*, *Dermacentor*, *Haemaphysalis*, *Hyalomma* and *Amblyomma*) [23–25], leading to the description of a novel family within *Rickettsiales*: “*Candidatus* Midichloriaceae” [26]. Interestingly, several other haematophagous arthropods have been shown to host *Midichloriaceae* [27, 28]. The detection of *Midichloriaceae* in different arthropod species, together with the lack of concordance between the phylogenies of *Midichloria* bacteria and those of their tick hosts, suggests that horizontal transfer of *Midichloriaceae* between haematophagous arthropod hosts can take place through trophic interactions [23, 29].

Multiple lines of evidence indicate that *Midichloriaceae* may infect mammals. For instance, DNA from *M. mitochondrii* has been detected in blood samples of roe deer (*Capreolus capreolus*), an important *I. ricinus* host [30], and of domestic animals (horses, cattle, sheep and dogs) exposed to ticks [31]. Moreover, tick-parasitized humans and dogs present circulating *Midichloria-*specific antibodies [31, 32]. These findings suggest that ticks can transmit *Midichloria* to their vertebrate hosts via the blood meal, similarly to other tick-borne bacteria such as *Borrelia* spp*.*, *Anaplasma* spp. and *Rickettsia* spp. [1]. This may be the case because *M. mitochondrii* occurs in *I. ricinus* salivary glands [32].

Seropositivity for *Midichloria* in tick-infested mammals indicates that the detection of *Midichloria* DNA in blood samples of vertebrates likely reflects a true *Midichloria* infection (involving bacterial replication within the host), resulting in immune response, with potentially negative effects on health state of the infected subjects [31]. Moreover, other studies have provided indications of pathogenicity or immunogenic effects to vertebrates caused by other members of *Midichloriaceae*. For instance, acute fever symptoms in humans exposed to tick parasitism have been attributed to “*Ca*. Lariskella arthropodarum”, and the pathogenic effects related to red mark syndrome in the rainbow trout, *Oncorhynchus mykiss*, have been associated with *Midichloria*-like bacteria [29, 33]. Despite these findings, the mechanisms of interaction between these bacteria and their vertebrate hosts are still poorly known. Although members of the *Midichloriaceae* have been found to circulate in mammals and fish, their frequency of occurrence and possible pathogenic effects in birds have yet to be assessed. Similarly, the role of avian hosts as natural reservoirs and potential vectors for these bacteria has never been investigated to date. This is surprising, considering that many bird species are common hosts of tick species which are known to harbour *Midichloriaceae*, such as those belonging to the genera *Ixodes* and *Hyalomma* [8, 34].

In this study, we focus on patterns of *Midichloria* infection in African ticks and in their trans-Saharan migratory avian hosts. We first screened for *Midichloria* DNA African ticks carried by trans-Saharan migratory songbirds sampled while crossing the Mediterranean Sea during their travel from sub-Saharan non-breeding grounds to European breeding quarters. We then assessed the extent to which these bacteria are transmitted by ticks to their avian hosts, by searching for *Midichloria* DNA in birds’ peripheral blood, and whether blood *Midichloria* presence was associated with tick parasitism. Finally, we investigated whether tick parasitism and blood *Midichloria* presence negatively affected fitness-related traits of avian hosts, by focusing on timing of migration and body condition indexes (amount of subcutaneous fat deposits, breast muscle thickness and body mass). In migratory birds, early arrival at the breeding grounds provides fitness benefits, because early arriving individuals face lower intraspecific competition and have better access to resources than late arriving ones, enjoying mating advantages [35–37]. However, only individuals in a prime health state can afford migrating and arriving early [35, 38, 39]. Thus, if ticks and/or *Midichloria* blood presence have pathogenic effects and negatively affect health and condition of avian hosts during migration, we predicted tick-infested and/or *Midichloria*-positive birds to have a poorer body condition (lower subcutaneous fat deposits, thinner breast muscles, lower body mass) and to delay their timing of migration (later capture date).

## Methods

### Bird and tick sampling protocols

Fieldwork was carried out on Ventotene Island (40°48′N, 13°26′E), an important stopover site in the Central Mediterranean Sea for migratory songbirds moving between Africa and Europe during spring migration. Most migratory birds reach the island after a non-stop crossing of the Mediterranean Sea [40, 41] and rest there for a few hours before resuming their northward journey [42]. Birds were captured by standardized mist-netting from April 8 to May 29, 2015, encompassing most of the migratory period of trans-Saharan migratory birds [41]. As a proxy for timing of migration, we considered the first capture date of each individual (i.e. we did not sample birds previously ringed during the same season on Ventotene) [43]. We focused on three trans-Saharan migratory passerine species [common redstart (*Phoenicurus phoenicurus*), whinchat (*Saxicola rubetra*) and common whitethroat (*Sylvia communis*); target species hereafter], which could be sampled in high numbers (300–1200 individuals/year) and had a high prevalence of tick-infested individuals (10–20%), based on previous surveys ([44]; J. G. Cecere, unpublished data). In addition, these target species were selected because the distinction between age (second-year *vs* > 2 years old, hereafter adult) and sex-classes according to plumage traits was feasible [45], allowing us to explore age- and sex-specific patterns of tick parasitism and blood *Midichloria* presence.

Upon capture and identification, birds were individually marked with metal rings and their sex (when feasible) and age (when feasible) were recorded. The amount of visible subcutaneous fat deposits (fat score; 0–8 scale; [46]) and the thickness of pectoral muscles (muscle score; 0–3 scale; [47]) were recorded as size-independent proxies for individual general state and body condition. Higher values of fat and muscle score during migration denote birds in a better health state (see [46, 48]). Body mass was also recorded (to the nearest 0.1 g) as a further body condition index. All individuals of target species were checked for the presence of ticks, systematically searching the ear canals, head, neck and eye surroundings. Ticks were also collected from trans-Saharan passerine migrants of non-target species whenever they were observed during standard ringing activities. Ticks were removed using O’tom tick twister® (H3D, Lavancia-Épercy, France) or Dumostar #3 Tweezers; all ticks collected from the same bird were then placed in microcentrifuge tubes containing 90% ethanol, and stored at room temperature for later identification. Blood samples were collected from all tick-infested birds of target species, as well as from a subset of birds without ticks at the moment of capture (‘non-infested birds’ hereafter). A small amount of blood (10–60 μl) was collected from the brachial vein into heparinized capillary tubes using sterile needles, and kept at *c*.4 °C before being stored at -80 °C for later analyses. In order to obtain about 30 blood samples from non-infested birds for each target species, the sampling rate of non-infested birds was set according to tick prevalence and capture data from previous years on the island (J. G. Cecere, unpublished data; see [49] for a similar procedure). Sampling rates were thus set as follows: common redstart, 1 non-infested individual out of 2 (*c.*13% tick parasitism and *c.*300 birds per season); whinchat, 1 out of 6 (*c*.16% tick parasitism and *c*.600 birds per season); common whitethroat, 1 out of 11 (*c*.8% tick parasitism and *c.*1200 birds per season). In addition, blood samples were collected from a subset of tick-infested birds of non-target passerine species, and for each of these individuals a blood sample was collected from a non-infested bird of the same species captured immediately afterwards. Hence, dyads of tick-infested and non-infested birds were established in order to compare the effects of tick parasitism and blood *Midichloria* presence on fitness-related traits of avian hosts. For non-target species from which we collected ticks, in a few cases we were unable to obtain blood samples from tick-infested individuals (e.g. *Phylloscopus sibilatrix*), or we were unable match a non-infested bird with a tick-infested one because of unpredictable migration fluxes.

### Morphological identification of tick specimens

All ticks were examined with a Leica MS5 stereomicroscope (Leica Microsystems GmbH, Wetzlar, Germany), categorized based on stage of development (larval, nymphal and adult), and identified to the species/genus level according to standard taxonomic keys [50, 51]. Though the vast majority of ticks could be morphologically identified to the species level, this was not possible for a few larvae (see Results). Although these unidentified specimens could theoretically be identified to the species level using molecular markers (e.g. [52, 53]), identification of all collected ticks to the species level was beyond the scope of our study.

### DNA extraction from ticks and blood samples

Total genomic DNA was extracted from the collected ticks and blood samples in order to detect *Midichloria* DNA. Stored tick specimens were rehydrated and washed twice in PBS 1× for 15 min, then left to dry for 20 min to remove all ethanol residues. Each specimen was placed in a 2 ml microcentrifuge tube and triturated with a scalpel blade into a solution containing 2 μl of proteinase K (20 mg/ml) and a specific volume of TRIS HCl (between 100 and 350 μl), depending on tick size. Blood samples were transferred from capillary tubes to microcentrifuge tubes, and an aliquot of blood (10–20 μl, depending on the available quantity of blood) was diluted with 90–130 μl of TE 1× and treated with 1 μl of proteinase K (20 mg/ml). The lysis was carried out with shaking at 56 °C overnight for tick specimens and for 30 min for blood samples; then, proteinase K activity was blocked at 90 °C for 5 min. Samples were subsequently centrifuged for 10 min at 20,000×*g*, stored at -20 °C and after use at − 80 °C to avoid DNA degradation.

### Molecular screening for *Midichloria* DNA in ticks and blood samples

*Midichloria* DNA in ticks and blood samples was screened using a modified version of the protocol described by [23], with two sets of primers targeting the bacterial *16S* rRNA gene. The first set of primers (Midi-F: 5′-GTA CAT GGG AAT CTA CCT TGC-3′; Midi-R: 5′-CAG GTC GCC CTA TTG CTT CTT T-3′; primers final concentration: 1 μM; amplification size: 1100 bp) was used for a first round of amplification. The second set of primers (Midi-F2: 5′-CAA AAG TGA AAG CCT TGG GC-3′; Midi-R2: 5′-TGA GAC TTA AAY CCC AAC ATC-3′) was used to perform a semi-nested PCR (Midi-F/Midi-R2, primers final concentration: 1 μM, amplification size: 691 bp) for tick samples and a nested PCR (Midi-F2/Midi-R2, primers final concentration: 1 μM, amplification size: 250 bp) for blood samples, respectively. Amplification was carried out using the following thermal profile: 30 s at 95 °C, 30 s at 57 °C and 45 s at 72 °C for 40 times.

### Sequence processing and analysis of *Midichloria* amplicons

PCR products were loaded on agarose gel, excised and purified with Wizard® SV Gel and PCR Clean-Up System (Promega, Milan, Italy), then sequenced by ABI technology (Applied Biosystems, Foster City, CA, USA). After manually correcting the electropherograms, sequences were subjected to BLAST analysis (https://blast.ncbi.nlm.nih.gov/). Representative long (> 1000 nt) sequences were added to a dataset of long (> 950 nt) publicly available *16S* rRNA *Midichloria* sequences detected in ticks and vertebrates. The sequences were aligned using MUSCLE software [54] and used to reconstruct a phylogenetic tree using the software PhyML Version 3.0, with 100 bootstraps [55]. Two *Cyrtobacter* sequences were used as outgroups.

### Effects of ticks and blood *Midichloria* presence on avian fitness-related traits

We first investigated whether the probability of hosting ticks among target species varied between sex (female *vs* male) and age (second-year *vs* adult) classes in a logistic regression, while including avian host species identity as a 3-level factor. All two-way interactions were included in the model. Then, the effects of tick parasitism on fitness-related traits of avian hosts (timing of migration, fat score, muscle score, and body mass) were investigated by linear models. Regarding target species, the effect of tick parasitism (tick-infested/non-infested) on timing of migration (capture date; day 1 = April 1) was tested in a model accounting for differences among species, by including avian host species identity as a 3-level factor. Sex and age were also included because they were expected to explain variation in timing of spring migration in these species [41, 56]. Since not all individuals of target species could be sexed and aged in the field, a smaller sample size was available for analyses involving sex and age effects than for those not controlling for sex and age. All two- and three-way interaction terms between species, sex and age were included in the initial model and then removed in a single step [three-way interaction first (if tested), then two-way ones] if non-significant (*P* > 0.05). Similar linear models were run to investigate the effects of tick parasitism on body condition indexes of avian hosts (fat score, muscle score and body mass). The effects of tick parasitism on body condition indexes were also analyzed on the entire set of non-target species by running a linear mixed model with avian host species identity and dyad as random intercept effects, and tick parasitism as a fixed effect factor. Sex and age effects were not considered in these models since they could not be determined in the field for several non-target species [45, 57].

To investigate the factors related to blood *Midichloria* presence in avian hosts we first tested whether the probability of detecting *Midichloria* in blood samples of target species was affected by tick parasitism at the time of capture. To this end, we ran a logistic regression analysis, where the binary dependent variable was blood *Midichloria* presence (0 = blood sample negative for *Midichloria* DNA; 1 = positive), while avian host species identity, tick parasitism, sex and age were the predictors, as well as all two-way interaction terms. The effects of blood *Midichloria* presence on fitness-related traits were then analyzed by running linear models and linear mixed models. Since blood *Midichloria* presence was tested on a subset of the large sample of individuals that was screened for tick parasitism (see Bird and tick sampling protocols), the size of the sample available for statistical analyses was considerably smaller than the sample of birds screened for ticks. To avoid model over-parametrization, for each fitness-related trait of target species we ran an initial linear model with the same fixed effect structure as the simplified models used to evaluate the effects of tick parasitism on the different fitness-related traits (see Results). Blood *Midichloria* presence was added to these models as a fixed effect factor, together with its two-way interactions with the other factors (species identity, tick parasitism, sex and age). Regarding non-target species, linear mixed models were carried out with body condition indexes as the dependent variables, tick parasitism, blood *Midichloria* presence and their interaction terms as the predictors, and avian host species identity and dyad as random intercept effects. Models were simplified by removing non-significant interaction terms in a single step. Linear mixed models were fitted using REML, and degrees of freedom were calculated according to the Kenward-Roger approximation. All statistical analyses were carried out using SAS® 9.3 software [58].

## Results

### Tick parasitism in trans-Saharan migratory birds

We found that 5.7% of the 1772 screened individuals belonging to the three target species was tick-infested (Table 1). We collected 259 ticks from 101 birds of target species, and 180 ticks from 68 tick-infested birds belonging to 13 non-target species (Table 1 and Additional file 1: Table S1). Tick specimens collected from target species were mostly immature stages (nymphs and larvae; a single adult was found) and the vast majority was identified either as belonging to the *H. marginatum* complex (226 nymphs) or to the genus *Hyalomma* (30 larvae) (Table 2). Only three ticks were identified as *Ixodes* spp. (Table 2). Similarly, most of the ticks removed from non-target species were immature stages of the *H. marginatum* complex (119 nymphs) or the genus *Hyalomma* (51 larvae). Eight ticks were identified as *Ixodes* spp. and two as *Haemaphysalis* spp. (Table 2 and Additional file 2: Table S2).

**Table 1:** Tick infestation on migratory birds of target species caught on Ventotene island (spring 2015) and number of collected ticks per host species

Avian host	No. of tick-infested/examined birds (%)	Infestation rate^a^ (95% CI)]	Min-max number of ticks/bird	No. of collected ticks
*Phoenicurus phoenicurus*	19/223 (8.5)	2.21 (1.33–3.68)	1–11	41
*Saxicola rubetra*	26/374 (7.0)	4.38 (3.01–6.39)	1–16	114
*Sylvia communis*	57/1175 (4.9)	1.82 (1.49–2.24)	1–7	104

^a^Number of ticks per tick-infested bird

The confidence intervals (95% CI) of the tick infestation rate were estimated according to an overdispersed Poisson distribution [68]

**Table 2:** Morphological identification of ticks collected from avian hosts of target and non-target species . See Additional file 1: Table S1 and Additional file 2: Table S2 for details about non-target species

Avian host	*Hyalomma* spp.	*H. marginatum* complex	*Ixodes* spp.	*Haemaphysalis* spp.
Target species				
*P. phoenicurus*	10	30	1	–
*S. rubetra*	10	104	–	–
*S. communis*	10	92	2	–
Non-target species				
13 species	51	119	8	2

### Molecular screening for *Midichloria* DNA in ticks and blood samples

Due to the very small sample of *Ixodes* and *Haemaphysalis* ticks, we focused on *Hyalomma* ticks only. All 426 *Hyalomma* ticks collected from both target and non-target species were tested for the presence of *Midichloria* DNA. The overall prevalence of *Midichloria* bacteria in ticks from target species was very high, being 93.8% in nymphs (212 out of 226) and 96.7% in larvae (29 out of 30) (Additional file 3: Table S3). Similar results were obtained for non-target species, where 96.6% of nymphs (115 out of 119) and 94.1% of larvae (48 out of 51) tested positive for *Midichloria* DNA (Additional file 3: Table S3). Rates of *Midichloria* presence in blood samples of tick-infested and non-infested birds were similar: *Midichloria* DNA was detected in 44.3% of blood samples (43 out of 97) from tick-infested birds and in 40.0% of blood samples (40 out of 100) from non-infested individuals of target species (Additional file 3: Table S3). Among non-target species, *Midichloria* DNA was detected in 46.4% of tick-infested birds’ blood samples (13 out of 28), and in 42.8% of non-infested birds’ blood samples (12 out of 28) (Additional file 3: Table S3).

### Phylogenetic analyses of *Midichloria* sequences

Direct sequencing of PCR products was performed for 19 *H. marginatum* tick specimens and 10 blood samples from four different bird species [the three target species plus the pied flycatcher (*Ficedula hypoleuca*); note that tick and blood sample PCR products were from different birds]. Long (> 990 nt) *16S* rRNA sequences were obtained after the first round of amplification from 5 out of 19 *H. marginatum* specimens, while shorter sequences were obtained from the other tick specimens after the second round of amplification (< 640 nt; see Additional file 4). For blood samples, we were able to obtain only partial sequences from nested protocols (see Additional file 4), as expected in the case of low bacterial load. BLAST analysis unambiguously identified all 29 sequences as *Midichloria*. This demonstrates the specificity of our results, in spite of the difficulties in amplification and sequencing of PCR products from blood samples. The representative longest sequence (1016 nt) of *Midichloria* from *H. marginatum* was deposited in GenBank (accession no. LT898326.1) and compared with two available *Midichloria* sequences from *H. marginatum* (GenBank: KY674395.1 and AM181354.1). The other 4 long sequences we obtained were identical to LT898326.1 (Additional file 5: Table S4; see also Additional file 4). Moreover, our novel sequence was identical to KY674395.1 (a very short sequence, 386 nt) and shared 98.2% sequence identity with AM181354.1 (Additional file 5: Table S4). Pairwise comparisons of all *Midichloria* sequences obtained from ticks, blood samples and AM181354.1 showed sequence identity values ranging between 98.1 and 100% (Additional file 5: Table S4; see also Additional file 4).

**Fig. 1:**
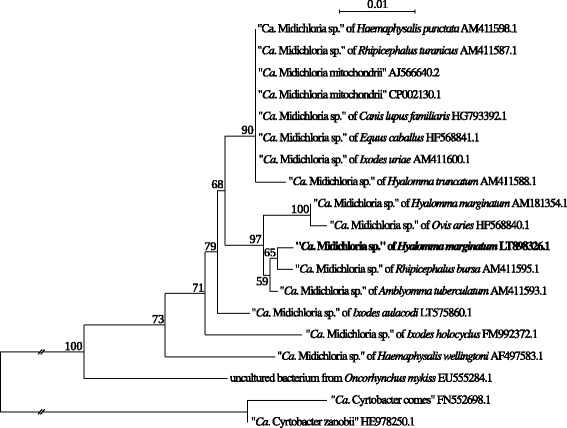
Phylogenetic tree of the genus *Midichloria*, obtained with Maximum Likelihood analysis of a *16S* rRNA gene alignment of long (> 950 nt) sequences. The representative sequence of *Midichloria* from *Hyalomma marginatum* obtained in this study (GenBank: LT898326.1) is highlighted in boldface. Bootstrap values above 50 are shown on the respective branches. *Scale-bar*: 1 nucleotide substitution per 100 positions. *Abbreviation*: “*Ca*.”, “*Candidatus*”

The phylogenetic reconstruction showed that our novel LT898326.1 sequence was located within the monophylum of *Midichloria* symbionts of ticks, as expected (Fig. 1) (see Additional file 6 for aligned sequences). The phylogenetic tree confirmed a lack of co-cladogenesis between symbionts and tick hosts, as previously reported [23]: the two long *Midichloria* sequences from *H. marginatum* (LT898326.1 and AM181354.1) were indeed located in separate branches of the tree.

### Effects of tick parasitism on avian host fitness-related traits

The probability of hosting ticks by target species did not significantly differ between age and sex classes, while accounting for inter-species differences in tick parasitism (Additional file 7: Table S5). Tick-infested individuals migrated through Ventotene slightly but significantly later than non-infested ones (*F*_(1,1326)_ = 4.65, *P* = 0.031), as highlighted by a linear model of timing of migration where species identity, age and sex differences in timing of migration were controlled for (Additional file 8: Table S6). Tick parasitism was associated with an estimated delay in timing of spring migration of 3.1 days (estimated mean values ± SE, non-infested individuals: 29.41 ± 0.41; tick-infested individuals: 32.48 ± 1.40). There were no significant differential effects of tick parasitism on timing of migration according to species identity, age and sex (see Additional file 8: Table S6 for details). There was no significant difference in body condition indexes between tick-infested and non-infested individuals, both in target and non-target species (Additional file 9: Table S7). Two-way interaction terms between tick parasitism and other factors were never statistically significant and were thus removed from all models (Additional file 9: Table S7).

### Effects of blood *Midichloria* presence on avian host fitness-related traits

**Fig. 2:**
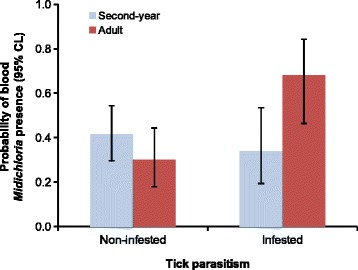
Probability of blood *Midichloria* DNA presence according to tick parasitism in adult and second-year individuals of target avian species. Tick-infested adult birds were significantly more likely to harbour *Midichloria* DNA in peripheral blood than non-infested adults and second-year birds at pairwise *post-hoc* tests (all *P* < 0.015; details not shown) (see Additional file 10: Table S8 for model details). Binomial 95% confidence limits were calculated using the ‘score method’ [69]

**Fig. 3:**
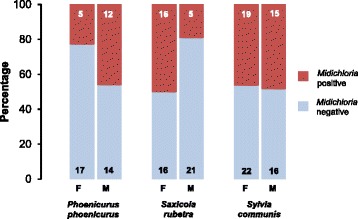
Percentage (%) of blood *Midichloria* DNA-positive individuals in the three target species according to host sex. Numbers within bars represent sample sizes. *Abbreviations*: M, males; F, females

**Fig. 4:**
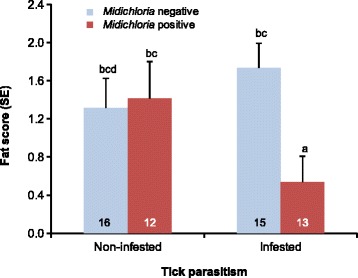
Fat score (mean + SE) of non-target avian host species according to tick parasitism and blood *Midichloria* DNA presence. Numbers within bars indicate sample size, while different letter combinations indicate statistically significant differences at *post-hoc* tests (a *vs* bc: *P* < 0.05; a *vs* bcd: *P* < 0.10; all other comparisons: *P* > 0.27)

In target species, tick-infested adults were significantly more likely to harbour *Midichloria* DNA than non-infested ones, whereas this was not the case among second-year individuals (logistic regression, tick parasitism × age, *χ*^2^ = 8.88, *df* = 1, *P* = 0.003; Fig. 2) (model details in Additional file 10: Table S8). Moreover, there were significant differences among species in blood *Midichloria* prevalence between males and females (species × sex, *χ*^2^ = 9.81, *df* = 2, *P* = 0.007): male common redstarts had a higher prevalence of *Midichloria* than females, while the opposite was the case in whinchats (Fig. 3). Male and female common whitethroats had similar rates of blood *Midichloria* presence (Fig. 3). There was no significant association between timing of migration of target species and blood *Midichloria* presence (Additional file 11: Table S9), as well as between body condition indexes and blood *Midichloria* presence (Additional file 12: Table S10). Two-way interactions involving blood *Midichloria* presence were never statistically significant and were thus removed from the models (Additional file 11: Table S9, Additional file 12: Table S10). Among non-target species, fat score was significantly lower in tick-infested individuals testing positive for blood *Midichloria* presence compared to non-infested birds and to tick-infested birds testing negative for blood *Midichloria* presence (mixed linear model, tick parasitism × blood *Midichloria* presence, *F*_(1,51.4)_ = 4.86, *P* = 0.032; Fig. 4). Other body condition indexes of non-target species were not significantly affected by blood *Midichloria* presence (Additional file 12: Table S10).

## Discussion

We found that *c.*5% of the trans-Saharan migratory birds belonging to the three target species (common redstart, whinchat and common whitethroat) harboured *Hyalomma* ticks of African origin while crossing the Mediterranean Sea during spring migration, while parasitism by other tick species was negligible. Almost all of the *Hyalomma* ticks collected in this study were nymphs, and about half of them were semi- or fully-engorged, suggesting that they had been attached on their hosts since 1–2 weeks. In fact, immature *H. marginatum* ticks have a long duration of attachment and feeding on the host owing to their two-host life-cycle [1, 59]. Due to the rapid spring movements of songbirds from Africa to Europe, these results are consistent with *Hyalomma* ticks mostly originating from sub-Saharan regions or North Africa [8, 59]. The rest of collected nymphs were unengorged and, since ixodid ticks do not start feeding until 24–36 h after attachment [1], they had probably just moulted from larvae on the host or they had attached to the host in North Africa, possibly within 36 h of their collection on Ventotene. Since none of the sampled migrants breed on the island and all of them are expected to continue their migration northward, our findings support previous suggestions that trans-Saharan migratory birds significantly contribute to the spread of African ticks and associated microbes in Europe while travelling from the non-breeding to the breeding grounds in spring [8, 59–61].

This is the first study assessing the presence of *Midichloria* bacteria in ticks parasitizing migratory songbirds and their occurrence in the blood of avian hosts. *Midichloria* prevalence in *Hyalomma* specimens was very high in both nymphs and larvae. Furthermore, DNA of *Midichloria* bacteria was detected in a considerable fraction of avian hosts’ blood samples (*c.*40%), with similar frequencies in tick-infested and non-infested birds. Hence, ticks may inoculate these bacteria in the avian bloodstream during the blood meal, as has been previously shown to occur in other tick hosts [31]. Since blood samples collected from non-infested birds were positive for *Midichloria* DNA, it may be hypothesized that these birds had undergone a previous infestation with ticks harbouring *Midichloria* and that the resulting presence of *Midichloria* had outlasted tick parasitism. The fact that presence of *Midichloria* was not strictly related to tick parasitism strongly suggests that it likely reflects live bacterial cells, which replicate and persist within vertebrate hosts, possibly triggering an antigenic response, as previously hypothesized [31, 32]. Further studies are required to assess the extent of bacterial replication within vertebrate hosts, as well as the role of birds in the transmission ecology of these bacteria, and the effectiveness of the avian host immune response in fending off the potentially ensuing infection.

PCR products obtained from the amplification of fragments of the *16S* rRNA gene of *Midichloria* from both ticks and blood samples were sequenced. Although the *16S* rRNA marker is not suitable for refined phylogenetic discriminations due to limited genetic variability, the lack of co-cladogenesis between bacteria and their tick hosts in the resulting tree further supports the hypothesis that *Midichloria* bacteria can transfer horizontally between hosts, possibly during the tick blood meal [23, 29].

Our findings also indicate that tick parasitism and blood *Midichloria* presence may exert detrimental effects on some fitness-related traits of avian hosts, and provide some indications on factors affecting the probability of having *Midichloria* DNA circulating in peripheral blood of trans-Saharan migratory birds.

First, we uncovered a delay in timing of spring migration across the Mediterranean Sea of tick-infested avian hosts, that migrated through Ventotene on average three days later than non-infested individuals. This finding is in accordance with previous investigations on the effect of parasite infections on timing of migration of avian hosts (e.g. [39, 62, 63]). Despite tick parasitism being associated with delayed migration, no significant differences in body condition emerged between tick-infested and non-infested individuals. Overall, our findings corroborate the idea that natural tick infestation levels do not heavily hamper avian hosts’ fitness [11, 12, 17, 18]. However, potential detrimental effects of ticks may be apparent on different health or physiological parameters of avian hosts than those considered here, such as immune system activation [11, 13], which may be linked to delayed migration. We envisage the following potential mechanism by which ticks may cause a delay in timing of migration: (i) depressing body condition of the avian hosts prior to the onset of migration, which could affect fat deposition rates; and (ii) causing physical exhaustion during flight, whereby tick-infested birds would need to spend more time on stopover sites to recover and rebuild fat deposits required to complete migration. Alternatively, the association between tick parasitism and timing of migration may reflect variation in individual quality, whereby low-quality individuals migrate later and are more likely to be parasitized by ticks than high-quality, early migrating ones [39].

Secondly, blood *Midichloria* presence negatively affected fat deposits of non-target species, which were lower in individuals hosting ticks and harbouring *Midichloria* DNA in peripheral blood compared to birds not infested by ticks, and to those individuals parasitized by ticks but not harbouring *Midichloria*. This finding suggests that possible pathogenic effects of blood *Midichloria* presence on vertebrate fitness-related traits may be subtle and emerge only in combination with other parasite effects. It should be emphasized that *Midichloria* effects may also be related to variation in the titer of bacteria among individual birds. Moreover, differences in the effects of blood *Midichloria* presence on fat deposits between target and non-target species suggest that species-specific traits may affect the sensitivity to *Midichloria* DNA.

Significant differences in blood *Midichloria* prevalence were also detected between males and females in two of the three target species. The origin of such interspecies differences between the sexes is unclear: they may either reflect sex differences in susceptibility to *Midichloria* infection due to differences in immunocompetence between the sexes [64, 65], or differences in behaviour between the sexes which may differentially affect the probability of *Midichloria* transmission by infected ticks.

## Conclusions

Our study expands current knowledge on the patterns of *Midichloria* infection among African ticks and on the interaction dynamics between ticks, tick-borne bacteria and their avian vertebrate hosts. We uncovered a high infection rate of *Midichloria* bacteria in tick specimens, and *Midichloria* DNA occurred in a remarkable *c*.40% of avian blood samples, irrespective of tick parasitism, indicating that these bacteria are effectively transmitted to avian hosts, that can widely circulate them across continents. Long-distance migratory birds may thus act as reservoirs for the worldwide spread of *Midichloria* across haematophagous ectoparasite populations. Future studies may investigate possible ‘hidden’ costs of blood *Midichloria* infection for vertebrate hosts (such as a reduction of lifespan or lifetime breeding success, e.g. [66]), as well as patterns of co-transmission of *Midichloria* with other known pathogenic bacteria*.* In fact, negative effects of tick parasitism and blood *Midichloria* presence on host fitness may also be partly explained by host co-infection with other pathogenic agents [67] that constitute the ‘package’ of antigens transmitted to vertebrate hosts during the tick blood meal.

## Additional files

Additional file 1: **Table S1.** Number of tick-infested avian hosts of non-target species and number of ticks collected from them. (DOCX 14 kb)

Additional file 2: **Table S2.** Morphological identification of tick specimens collected from birds of non-target species. (DOCX 15 kb)

Additional file 3: **Table S3.** Patterns of *Midichloria* DNA detection in *Hyalomma* tick specimens and blood samples collected from trans-Saharan migratory birds. (DOCX 16 kb)

Additional file 4: Aligned *Midichloria* sequences obtained in this study (FASTA). (TXT 31 kb)

Additional file 5: Aligned *Midichloria* sequences used for the phylogenetic reconstruction (FASTA). (TXT 28 kb)

Additional file 6: **Table S4.** Pairwise matrix showing the % sequence identity between pairs of *16S* rRNA *Midichloria* sequences obtained in this study and AM181354.1. (DOCX 15 kb)

Additional file 7: **Table S5.** Logistic regression exploring the effects of species identity, sex and age on tick parasitism. (DOCX 14 kb)

Additional file 8: **Table S6.** Linear model of the effects of tick parasitism on timing of migration of avian hosts of target avian hosts. (DOCX 14 kb)

Additional file 9: **Table S7.** Models of the effects of tick parasitism on body condition indexes of target and non-target avian hosts. (DOCX 16 kb)

Additional file 10: **Table S8.** Logistic regression of the effects of tick parasitism on blood *Midichloria* DNA presence of target avian hosts. (DOCX 14 kb)

Additional file 11; **Table S9.** Linear model of the effect of blood *Midichloria* DNA presence on timing of migration of individuals of target avian host species. (DOCX 14 kb)

Additional file 12: **Table S10.** Models of the effect of blood *Midichloria* DNA presence on body condition indexes of target and non-target avian host species. (DOCX 17 kb)
