# Supplementary material for: Patterns of Midichloria infection in avian-borne African ticks and their trans-Saharan migratory hosts
Source: Parasit Vectors. 2018 Feb 22;11:106. doi: 10.1186/s13071-018-2669-z (PMC5824480; doi:10.1186/s13071-018-2669-z)
Supplement: Supplementary file 1 — Table S1. Number of tick-infested avian hosts of non-target species and number of ticks collected from them. (DOCX 14 kb) [file 13071_2018_2669_MOESM1_ESM.docx]

Table S1. Number of tick-infested avian hosts of non-target species and number of ticks collected from them.

| **Avian host** | **No. tick-infested birds** | **No. collected ticks** |
| --- | --- | --- |
| *Acrocephalus schoenobaenus* | 4 | 10 |
| *Anthus trivialis* | 2 | 2 |
| *Ficedula albicollis* | 2 | 3 |
| *Ficedula hypoleuca* | 8 | 20 |
| *Hippolais icterina* | 5 | 20 |
| *Luscinia megarhynchos* | 13 | 25 |
| *Motacilla flava* | 4 | 11 |
| *Muscicapa striata* | 6 | 14 |
| *Oenanthe hispanica* | 1 | 1 |
| *Oenanthe oenanthe* | 4 | 30 |
| *Oriolus oriolus* | 8 | 26 |
| *Phylloscopus sibilatrix* | 5 | 12 |
| *Sylvia borin* | 6 | 6 |
| Total | 68 | 180 |
