# Supplementary material for: Patterns of Midichloria infection in avian-borne African ticks and their trans-Saharan migratory hosts
Source: Parasit Vectors. 2018 Feb 22;11:106. doi: 10.1186/s13071-018-2669-z (PMC5824480; doi:10.1186/s13071-018-2669-z)
Supplement: Supplementary file 2 — Table S2. Morphological identification of tick specimens collected from birds of non-target species. (DOCX 15 kb) [file 13071_2018_2669_MOESM2_ESM.docx]

Table S2. Morphological identification of tick specimens collected from birds of non-target species.

| **Avian host** | ***H. marginatum* complex** | ***Hyalomma* spp.** | ***Ixodes* spp.** | ***Haemaphysalis* spp.** |
| --- | --- | --- | --- | --- |
| *A. schoenobaenus* | 3 | 7 | - | - |
| *A. trivialis* | - | - | 1 | 1 |
| *F. albicollis* | - | 3 | - | - |
| *F. hypoleuca* | 17 | 2 | - | 1 |
| *H. icterina* | 11 | 9 | - | - |
| *L. megarhynchos* | 17 | 3 | 5 | - |
| *M. flava* | 11 | - | - | - |
| *M. striata* | 5 | 9 | - | - |
| *O. hispanica* | 1 | - | - | - |
| *O. oenanthe* | 22 | 8 | - | - |
| *O. oriolus* | 23 | 2 | 1 | - |
| *P. sibilatrix* | 7 | 5 | - | - |
| *S. borin* | 2 | 3 | 1 | - |
| Total | 119 | 51 | 8 | 2 |
