# Supplementary material for: Patterns of Midichloria infection in avian-borne African ticks and their trans-Saharan migratory hosts
Source: Parasit Vectors. 2018 Feb 22;11:106. doi: 10.1186/s13071-018-2669-z (PMC5824480; doi:10.1186/s13071-018-2669-z)
Supplement: Supplementary file 3 — Table S3. Patterns of Midichloria DNA detection in Hyalomma tick specimens and blood samples collected from trans-Saharan migratory birds. (DOCX 16 kb) [file 13071_2018_2669_MOESM3_ESM.docx]

Table S3. Patterns of *Midichloria* DNA detection in *Hyalomma* tick specimens and blood samples collected from trans-Saharan migratory birds. It was not possible to successfully collect blood samples from *P. sibilatrix*, which was therefore not included in the statistical analysis. No *Hyalomma* ticks were found on *A. trivialis*.

| **Avian host** | **No. positive larvae/collected (%)** | **No. positive nymphs/collected (%)** | **No. positive blood samples/collected from tick-infested birds (%)** | **No. positive blood samples/collected from non-infested birds (%)** |
| --- | --- | --- | --- | --- |
| *Target species* |  |  |  |  |
| *P. phoenicurus* | 9/10 (90.0%) | 30/30 (100.0%) | 6/18 (33.3%) | 13/33 (39.4%) |
| *S. rubetra* | 10/10 (100.0%) | 102/104 (98.0%) | 6/25 (24.0%) | 15/33 (45.5%) |
| *S. communis* | 10/10 (100.0%) | 80/92 (87.0%) | 31/54 (57.4%) | 12/34 (35.0%) |
| *Non-target species* |  |  |  |  |
| *A. schoenobaenus* | 7/7 | 3/3 | 0/1 | 1/1 |
| *A. trivialis* | - | - | 1/1 | 0/1 |
| *F. albicollis* | 3/3 | - | 1/1 | 1/1 |
| *F. hypoleuca* | 2/2 | 16/17 | 1/2 | 0/2 |
| *H. icterina* | 9/9 | 11/11 | 1/3 | 1/3 |
| *L. megarhynchos* | 3/3 | 17/17 | 3/8 | 1/6 |
| *M. flava* | - | 11/11 | 1/2 | 2/3 |
| *M. striata* | 7/9 | 5/5 | 2/2 | 2/3 |
| *O. hispanica* | - | 1/1 | 1/1 | 0/1 |
| *O. oenanthe* | 7/8 | 19/22 | 0/2 | 1/2 |
| *O. oriolus* | 2/2 | 23/23 | 1/4 | 3/4 |
| *P. sibilatrix* | 5/5 | 7/7 | - | - |
| *S. borin* | 3/3 | 2/2 | 1/1 | 0/1 |
| All species | 48/51 (94.1%) | 115/119 (96.6%) | 13/28 (46.4%) | 12/28 (42.8%) |
