# Supplementary material for: Patterns of Midichloria infection in avian-borne African ticks and their trans-Saharan migratory hosts
Source: Parasit Vectors. 2018 Feb 22;11:106. doi: 10.1186/s13071-018-2669-z (PMC5824480; doi:10.1186/s13071-018-2669-z)
Supplement: Supplementary file 5 — Aligned Midichloria sequences used for the phylogenetic reconstruction (FASTA). (TXT 28 kb) [file 13071_2018_2669_MOESM5_ESM.docx]

Table S4. Pairwise matrix showing the % sequence identity between pairs of 16S rRNA *Midichloria* sequences obtained in this study and AM181354.1, a long *Midichloria* sequence from *Hyalomma marginatum* retrieved from GenBank. SC22, SC136 and SR2 are short *Midichloria* sequences from blood samples of *Sylvia communis* and *Saxicola rubetra*, respectively, while LT898326.1 is the GenBank accession number of the representative longest (1016 nt) *Midichloria* sequence from *H. marginatum* obtained in this study. All the other sequences obtained in this study (i.e. 18 other sequences from *Hyalomma marginatum* and 7 other sequences from bird blood) showed 100% sequence identity with LT898326.1.

|  | AM181354.1 | LT898326.1 | SC22 | SC136 |
| --- | --- | --- | --- | --- |
| LT898326.1 | 98.2% | - |  |  |
| SC22 | 98.2% | 98.8% | - |  |
| SC136 | 98.7% | 99.6% | 98.8% | - |
| SR2 | 99.0% | 99.5% | 98.1% | 99.0% |
