# Supplementary material for: Patterns of Midichloria infection in avian-borne African ticks and their trans-Saharan migratory hosts
Source: Parasit Vectors. 2018 Feb 22;11:106. doi: 10.1186/s13071-018-2669-z (PMC5824480; doi:10.1186/s13071-018-2669-z)
Supplement: Supplementary file 7 — Table S5. Logistic regression exploring the effects of species identity, sex and age on tick parasitism. (DOCX 14 kb) [file 13071_2018_2669_MOESM7_ESM.docx]

Table S5. Logistic regression exploring the effects of species identity, sex and age on tick parasitism (probability of hosting ticks). All two-way interaction terms were non-significant and were removed from the model (p-values always > 0.26; details not shown for brevity).

| **Effect** | **χ2** | **df** | **p** |
| --- | --- | --- | --- |
| Bird species | 1.729 | 2 | 0.42 |
| Sex | 0.092 | 1 | 0.76 |
| Age | 2.241 | 1 | 0.13 |
