# Supplementary material for: Patterns of Midichloria infection in avian-borne African ticks and their trans-Saharan migratory hosts
Source: Parasit Vectors. 2018 Feb 22;11:106. doi: 10.1186/s13071-018-2669-z (PMC5824480; doi:10.1186/s13071-018-2669-z)
Supplement: Supplementary file 8 — Table S6. Linear model of the effects of tick parasitism on timing of migration of avian hosts of target avian hosts. (DOCX 14 kb) [file 13071_2018_2669_MOESM8_ESM.docx]

Table S6. Linear model of the effects of tick parasitism on timing of migration of target avian hosts. Non-significant two-way interaction terms between tick parasitism and other model factors were removed in a single step (all p-values > 0.44; details not shown for brevity).

| **Effect** | **F** | **df** | **p** |
| --- | --- | --- | --- |
|  |  |  |  |
| Bird species | 80.72 | 2, 1326 | < 0.001 |
| Tick parasitism | 4.65 | 1, 1326 | 0.031 |
| Sex | 122.46 | 1, 1326 | < 0.001 |
| Age | 8.89 | 1, 1326 | 0.003 |
| Bird species × Sex | 25.41 | 2, 1326 | < 0.001 |
| Bird species × Age | 7.18 | 2, 1326 | < 0.001 |
| Sex × Age | 1.07 | 1, 1326 | 0.30 |
| Bird species × Sex × Age | 6.58 | 2, 1326 | 0.001 |
