# Supplementary material for: Patterns of Midichloria infection in avian-borne African ticks and their trans-Saharan migratory hosts
Source: Parasit Vectors. 2018 Feb 22;11:106. doi: 10.1186/s13071-018-2669-z (PMC5824480; doi:10.1186/s13071-018-2669-z)
Supplement: Supplementary file 9 — Table S7. Models of the effects of tick parasitism on body condition indexes of target and non-target avian hosts. (DOCX 16 kb) [file 13071_2018_2669_MOESM9_ESM.docx]

Table S7. Models of the effects of tick parasitism on body condition indexes of target and non-target avian hosts. Two-way interaction terms between tick parasitism and other model factors were removed from final models since they were never significant (in all cases p-values were > 0.47; details not shown for brevity). Statistics for target species were obtained from linear models, whereas those for non-target species were derived from linear mixed models (see Methods for details).

| **Body condition indexes** | **Effect** | **F** | **df** | **p** |
| --- | --- | --- | --- | --- |
| *Target species* |  |  |  |  |
| Fat score | Bird species | 8.45 | 2, 1323 | < 0.001 |
|  | Tick parasitism | 0.17 | 1, 1323 | 0.68 |
|  | Sex | 2.73 | 1, 1323 | 0.10 |
|  | Age | 10.32 | 1, 1323 | 0.001 |
|  |  |  |  |  |
| Muscle score | Bird species | 34.46 | 2, 1333 | < 0.001 |
|  | Tick parasitism | 0.79 | 1, 1333 | 0.37 |
|  | Sex | 0.30 | 1, 1333 | 0.58 |
|  | Age | 5.95 | 1, 1333 | 0.015 |
|  |  |  |  |  |
| Body mass | Bird species | 79.06 | 2, 1323 | < 0.001 |
|  | Tick parasitism | 0.18 | 1, 1323 | 0.67 |
|  | Sex | 22.60 | 1, 1323 | < 0.001 |
|  | Age | 15.98 | 1, 1323 | < 0.001 |
|  | Bird species × Sex | 6.58 | 2, 1323 | 0.001 |
|  | Bird species × Age | 3.95 | 2, 1323 | 0.019 |
| *Non-target species* |  |  |  |  |
| Fat score | Tick parasitism | 2.01 | 1, 60 | 0.16 |
| Muscle score | Tick parasitism | 0.03 | 1, 60 | 0.87 |
| Body mass | Tick parasitism | 0.01 | 1, 109 | 0.95 |
