# Supplementary material for: Patterns of Midichloria infection in avian-borne African ticks and their trans-Saharan migratory hosts
Source: Parasit Vectors. 2018 Feb 22;11:106. doi: 10.1186/s13071-018-2669-z (PMC5824480; doi:10.1186/s13071-018-2669-z)
Supplement: Supplementary file 10 — Table S8. Logistic regression of the effects of tick parasitism on blood Midichloria DNA presence of target avian hosts. (DOCX 14 kb) [file 13071_2018_2669_MOESM10_ESM.docx]

Table S8. Logistic regression of the effects of tick parasitism on blood *Midichloria* DNA presence of target avian hosts. Non-significant two-way interaction terms between tick parasitism and other model factors were removed in a single step (all p-values > 0.40; details not shown for brevity).

| **Effect** | **χ2** | **df** | **p** |
| --- | --- | --- | --- |
| Bird species | 7.32 | 2 | 0.026 |
| Tick parasitism | 1.82 | 1 | 0.18 |
| Sex | 0.68 | 1 | 0.41 |
| Age | 0.35 | 1 | 0.56 |
| Bird species × Sex | 9.81 | 2 | 0.007 |
| Tick parasitism × Age^a^ | 8.88 | 1 | 0.003 |
| a: age-specific parameter estimates for the effect of tick parasitism:  adult, 1.72 (0.66 SE), z = 2.59, p = 0.010  second-year, -0.59 (0.44 SE), z = 1.33, p = 0.18 | | | |
