# Supplementary material for: Patterns of Midichloria infection in avian-borne African ticks and their trans-Saharan migratory hosts
Source: Parasit Vectors. 2018 Feb 22;11:106. doi: 10.1186/s13071-018-2669-z (PMC5824480; doi:10.1186/s13071-018-2669-z)
Supplement: Supplementary file 11 — Table S9. Linear model of the effect of blood Midichloria DNA presence on timing of migration of individuals of target avian host species. (DOCX 14 kb) [file 13071_2018_2669_MOESM11_ESM.docx]

Table S9. Linear model of the effect of blood *Midichloria* DNA presence on timing of migration of individuals of target avian host species. Non-significant two-way interaction terms between blood *Midichloria* DNA presence and other model factors were removed in a single step (all p-values > 0.22; details not shown for brevity), as well as other non-significant interactions (Bird species × Sex × Age, Species × Age and Sex × Age; details not shown for brevity).

| **Effect** | **F** | **df** | **p** |
| --- | --- | --- | --- |
| Bird species | 19.46 | 2, 151 | < 0.001 |
| Tick parasitism | 3.00 | 1, 151 | 0.09 |
| Blood *Midichloria* presence | 0.25 | 1, 151 | 0.62 |
| Sex | 36.16 | 1, 151 | < 0.001 |
| Age | 3.55 | 1, 151 | 0.06 |
| Bird species × Sex | 9.57 | 2, 151 | < 0.001 |
