# Supplementary material for: Patterns of Midichloria infection in avian-borne African ticks and their trans-Saharan migratory hosts
Source: Parasit Vectors. 2018 Feb 22;11:106. doi: 10.1186/s13071-018-2669-z (PMC5824480; doi:10.1186/s13071-018-2669-z)
Supplement: Supplementary file 12 — Table S10. Models of the effect of blood Midichloria DNA presence on body condition indexes of target and non-target avian host species. (DOCX 17 kb) [file 13071_2018_2669_MOESM12_ESM.docx]

Table S10. Models of the effect of blood *Midichloria* DNA presence on body condition indexes of target and non-target avian host species. Two-way interaction terms between blood *Midichloria* DNA presence and other model factors were removed from final models as they were never significant (in all cases p-values were > 0.12; details not shown for brevity). The starting models for these analyses were those reported in Table S7. Statistics for target species were obtained from linear models, whereas those for non-target species were derived from linear mixed models (see Methods for details).

| **Body condition indexes** | **Effect** | **F** | **df** | **p** |
| --- | --- | --- | --- | --- |
| *Target species* |  |  |  |  |
| Fat score | Bird species | 2.69 | 2, 153 | 0.07 |
|  | Tick parasitism | 0.36 | 1, 153 | 0.55 |
|  | Blood *Midichloria* presence | 1.51 | 1, 153 | 0.22 |
|  | Sex | 0.13 | 1, 153 | 0.72 |
|  | Age | 1.33 | 1, 153 | 0.25 |
|  |  |  |  |  |
| Muscle score | Bird species | 5.88 | 2, 153 | 0.004 |
|  | Tick parasitism | 0.10 | 1, 153 | 0.75 |
|  | Blood *Midichloria* presence | 0.00 | 1, 153 | 0.97 |
|  | Sex | 0.07 | 1, 153 | 0.80 |
|  | Age | 2.61 | 1, 153 | 0.11 |
|  |  |  |  |  |
| Body mass | Bird species | 7.03 | 2, 152 | 0.001 |
|  | Tick parasitism | 0.91 | 1, 152 | 0.34 |
|  | Blood *Midichloria* presence | 1.74 | 1, 152 | 0.19 |
|  | Sex | 1.92 | 1, 152 | 0.17 |
|  | Age | 0.40 | 1, 152 | 0.53 |
|  |  |  |  |  |
| *Non-target species* |  |  |  |  |
| Fat score | Tick parasitism | 0.61 | 1, 45.6 | 0.44 |
|  | Blood *Midichloria* presence | 2.29 | 1, 50.6 | 0.14 |
|  | Tick parasitism × Blood *Midichloria* | 4.86 | 1, 51.4 | 0.032 |
|  |  |  |  |  |
| Muscle score | Tick parasitism | 0.56 | 1, 28 | 0.46 |
|  | Blood *Midichloria* presence | 1.27 | 1, 45.5 | 0.27 |
|  |  |  |  |  |
| Body mass | Tick parasitism | 0.25 | 1, 26.8 | 0.62 |
|  | Blood *Midichloria* presence | 0.07 | 1, 40.2 | 0.79 |
